# Operational Issues and Trends Associated with the Pilot Introduction of Zinc for Childhood Diarrhoea in Bougouni District, Mali

**Published:** 2008-06

**Authors:** Peter J. Winch, Kate E. Gilroy, Seydou Doumbia, Amy E. Patterson, Zana Daou, Adama Diawara, Eric Swedberg, Robert E. Black, Olivier Fontaine, Mali Zinc Pilot Intervention Study Group

**Affiliations:** ^1^Department of International Health, Bloomberg School of Public Health, Johns Hopkins University, Baltimore, MD, USA; ^2^Faculty of Medicine/Department of Public Health, University of Bamako, Mali; ^3^Department of Behavioral Sciences and Health Education, Rollins School of Public Health, Emory University, Atlanta, Georgia, USA; ^4^Save the Children, Bougouni, Mali and Westport, Connecticut; ^5^Department of Child and Adolescent Health and Development, World Health Organization, Geneva, Switzerland (Members of the Mali Zinc Pilot Intervention Study Group are: Robert E. Black, Fatoumata Bouaré, Seyon Coulibaly, Maureen Cunningham, Zana Daou, Adama Diawara, Seydou Doumbia, Olivier Fontaine, Kate E. Gilroy, Danaya Koné, Soungalo Koné, Fodié Maguiraga, Rita Nader, Drissa Ouattara, Amy E. Patterson, Eric Swedberg, and Peter J. Winch)

**Keywords:** Antibiotic use, Diarrhoea, Child health, Oral rehydration solutions, Oral rehydration therapy, Zinc, Zinc therapy, Mali

## Abstract

Zinc for the treatment of childhood diarrhoea was introduced in a pilot area in southern Mali to prepare for a cluster-randomized effectiveness study and to inform policies on how to best introduce and promote zinc at the community level. Dispersible zinc tablets in 14-tablet blister packs were provided through community health centres and drug kits managed by community health workers (CHWs) in two health zones in Bougouni district, Mali. Village meetings and individual counselling provided by CHWs and head nurses at health centres were the principal channels of communication. A combination of methods were employed to (a) detect problems in communication about the benefits of zinc and its mode of administration; (b) identify and resolve obstacles to implementation of zinc through existing health services; and (c) describe household-level constraints to the adoption of appropriate home-management practices for diarrhoea, including administration of both zinc and oral rehydration solution (ORS). Population-based household surveys with caretakers of children sick in the previous two weeks were carried out before and four months after the introduction of zinc supplementation. Household follow-up visits with children receiving zinc from the health centres and CHWs were conducted on day 3 and 14 after treatment for a subsample of children. A qualitative process evaluation also was conducted to investigate operational issues. Preliminary evidence from this study suggests that the introduction of zinc does not reduce the use of ORS and may reduce inappropriate antibiotic use for childhood diarrhoea. Financial access to treatments, management of concurrent diarrhoea and fever, and high use of unauthorized drug vendors were identified as factors affecting the effectiveness of the intervention in this setting. The introduction of zinc, if not appropriately integrated with other disease-control strategies, has the potential to decrease the appropriate presumptive treatment of childhood malaria in children with diarrhoea and fever in malaria-endemic areas.

## INTRODUCTION

Diarrhoea is estimated to cause 18% of the estimated 10.6 million annual deaths in children, aged less than five years (under-five children), globally (1). Meta-analyses and reviews of previous trials have demonstrated that 10–14 days of zinc treatment is associated with decreased severity and duration of the diarrhoea episode and lower incidence of diarrhoea and pneumonia during two months following treatment, leading to decreased overall mortali-ty of children aged less than five years (under-five mortality) (2-4). Based on these findings, the World Health Organization (WHO) and the United Nations Children's Fund (UNICEF) currently recommend incorporating zinc into the diarrhoea-management guidelines for children aged under five years (5). As countries revise their national diarrhoea-treatment guidelines, the feasibility of incorporating zinc into the routine management of diarrhoea at the local level needs to be carefully examined (2,6,7). Only one study, in Bangladesh, has assessed the effectiveness of zinc as a treatment for diarrhoea in a large-scale community effectiveness trial (8,9). Studies investigating the feasibility of the introduction of zinc have recently concluded in Pakistan, India, and Mali, and preliminary results from formative research and pilot-test phases of these studies have been published (10-12). Finally, zinc supplementation for diarrhoea is being incorporated into the routine programmes on a large-scale in Bangladesh, and this roll-out is being carefully monitored (7,13).

One of the major concerns when introducing zinc into existing diarrhoea-management practices in the home is the potential that health providers or families might consider zinc a substitute for oral rehydration therapy (ORT), including oral rehydration salts (ORS) and appropriate home-fluids. Zinc is a new product in a ‘fancy' blister pack and may be a more attractive purchase than an ORS sachet, and parents often prefer to use modern treatments, which generally come in the form of pills or injections, to ‘cure' diarrhoea (14). Despite these concerns, findings from Matlab, Bangladesh, and preliminary findings from India suggest that the promotion of zinc supplementation may increase the use of ORS for uncomplicated diarrhoea and decrease the unnecessary use of antibiotics (8,10).

This paper describes the results of a pilot study in southern Mali in which various quantitative and qualitative methods were employed to identify problems relating to the introduction of this new intervention and suggest ways to refine the intervention package prior to any large-scale implementation. The impact and feasibility of large-scale implementation of this intervention is currently being assessed in a subsequent phase of research that started in October 2005. Rather than a formal effectiveness study, the main objectives of this pilot study were to: (a) evaluate the community promotion of zinc treatment and identify more effective channels of communication; (b) identify and resolve obstacles to implementation of zinc through community health centres and through a system of village drug kits managed by community health workers (CHWs); and (c) identify factors that may facilitate or impede the adoption of appropriate home management of diarrhoea, including supplementation with zinc.

## MATERIALS AND METHODS

### Study site

The study was carried out in two health zones in Bougouni district, located in southern Mali. Study participants mostly belong to the Bambara ethnic group, speak Bambara in the home, and reside in large compounds whose members belong to the same extended family. Each of these health zones has a population of 12,000–17,000 and is served by one community health centre (equivalent to health posts or dispensaries in other African countries) and a network of village drug kits managed by CHWs. Each health zone has approximately 8–10 village drug kits located in larger villages (>500 inhabitants) that are located five km or further from the health centre. The village drug kits contain products for common illnesses in the community, including antimalarials, paracetamol, and ORS. The CHW maintains these medications in a wooden cabinet in his or her household within the community. The CHWs attend supervisory meetings and restock their medications at the community health centre.

### Methods of intervention

Zinc was provided through the community health centres and the village drug kits managed by the CHWs. The health centre staff and CHWs were trained on (a) diarrhoea case management with zinc and ORS, (b) counselling of parents on child-feeding and prevention and treatment of diarrhoea, and (c) recording routine data in notebooks. Zinc was distributed in a 14-count blister package of dispersible tablets. The recommended regimen consisted of one 20-mg tablet per day for children aged six months or older and half a tablet, or 10 mg per day, for children aged less than six months (5).

Zinc was introduced through the existing distribution system for medications, based on the Bamako Initiative, which involves recovery of costs at each level (15-17). At the community health centres, villagers are charged a consultation fee and also pay separately for any prescriptions obtained—profits are, however, used for paying salaries of staff, maintaining the centre and equipment, and restocking supplies and medications. The CHWs volunteer their services, but charge for medications to replenish their stocks in the drug kits and cover additional operational costs. In principle, any additional revenues from the drug kits are split between the CHW and village oversight committees as an incentive for their continuing work. However, lack of adequate incentives for the CHWs is an ongoing challenge in this programme. The cost of zinc to patients at the health centres and CHWs was 100 CFA Francs (~0.19 US$) for the full package of 14 tablets and 50 CFA Francs (~0.09 US$) for a half blister package of seven tablets (for children aged less than 6 months). Zinc supplies were stored at the District Drug Warehouse at the referral hospital in Bougouni until purchased by the community health centres, which, in turn, supplied the CHWs. At the start of the intervention, the community health centres and CHWs were given a zinc supply estimated to last for two months. This supply chain for zinc was largely effective during the brief period of the pilot introduction, and no stock-outs were reported. However, the system is especially vulnerable to stock-outs at the CHW level, and stock-outs of ORS packets did occur in the community.

The promotion of zinc supplementation for the pilot intervention was done primarily through village meetings and individual counselling provided by the CHWs and head nurses at the health centres. The CHWs also promoted zinc treatment at community events, such as baptisms, marriages, and religious holidays. Key messages and visual aids were developed based on findings of a formative phase of research (11); they were used primarily by the project field supervisor during community meetings.

### Evaluation methods

Various methods were used for examining operational difficulties encountered in the pilot implementation of this intervention; these methods are briefly described here.

*Population-based household surveys:* Population-based household surveys with caretakers of children sick in the previous two weeks were carried out before (March-April 2004) and four months after the introduction of zinc supplementation (September 2004). Lists of all the housing compounds in each village located in the two pilot health zones and their approximate size were compiled. Housing compounds were chosen proportional to their size using a systematic sampling scheme. If there were two or more sick children residing in the compound, the interviewer drew the name of one of the children through lottery. If there was no sick child or the caretaker did not consent, the compound was replaced with the housing compound to the right. The surveys gathered data on: (a) treatment practices for diarrhoea, including the use of ORS, antibiotics, and zinc; (b) care-seeking from both health facilities and alternate providers; and (c) knowledge and use of zinc supplementation. Interviewers were initially trained for five days, with a three-day refresher training before the final survey. The baseline and final questionnaires, translated from French into Bambara collaboratively by the study team and interviewers, were pretested in an adjoining health zone and modified accordingly. In total, 352 surveys at baseline and 351 final surveys were carried out.

*Household follow-up visits:* Household follow-up visits with children receiving zinc from the health centres and CHWs were conducted on day 3 and 14 after treatment for a subsample of children. These surveys collected information describing the administration of zinc, purchasing patterns, compliance, attitudes, and caregiver satisfaction and monitored for adverse outcomes or side-effects of zinc, such as vomiting, convulsions, or death. This component of the study is described in more detail elsewhere (12).

*Process evaluation through collection of qualitative data:* A three-person team not involved in the intervention implementation conducted the process evaluation through collection of qualitative data. Interviews with the CHWs managing village drug kits were carried out to obtain information about the overall implementation of the intervention, including: training, supervision, community promotion, the use of zinc, management of diarrhoea, and the operational aspects of the CHW system (n=28). Semi-structured interviews were conducted with mothers, fathers, and other caretakers of young children regarding care-seeking and home management for childhood diarrhoea, knowledge and perceptions of the zinc intervention activities, knowledge and perceptions of the village drug kit system, and financial access to zinc and other medications. Interviewees were selected to include both families using zinc (n=28) and those not using zinc (and/or ORS) (n=9). CHW training sessions (n=3) and community meetings (n=13) promoting improved management of diarrhoea were directly observed and carefully documented. Information about children treated for diarrhoea was also collected and compiled from registers in the community health centres and CHW records (notebooks) through regular monitoring activities.

Because of the variety of methods employed, when quantitative data are cited in the text or tables, the data-collection method and sample size are indicated. This pilot introduction was not powered to detect differences between baseline and final surveys; however, we present these data to demonstrate observed trends associated with the introduction of zinc.

### Ethical approval

The study received ethical approval from the Committee for Human Research of Johns Hopkins University Bloomberg School of Public Health and from the Internal Review Board of the Faculty of Medicine, Pharmacy, and Dentistry, University of Bamako. Consent in each participating village was obtained from village leaders, and parents were asked for consent prior to interviews.

## RESULTS

### Awareness and use of zinc supplementation

Reaction to zinc supplements as a diarrhoea treatment was largely positive, and community members appreciated the packaging, the taste of the tablets, and the outcomes seen in their children post-administration. Community perceptions of zinc treatment were presented in more detail elsewhere (11,12). The promotion of zinc and improved management of diarrhoea was carried out primarily through community meetings conducted by the CHWs in their villages. In the final household survey conducted four months after the introduction of zinc, only 99 (28%) of 351 caretakers interviewed in the two health zones had any knowledge of zinc; however, 81 (59% of 137caretakers of sick children who lived in villages with a CHW had knowledge of zinc.

The reported use of zinc for children sick with diarrhoea during the previous two weeks was relatively low (17%), four months after the introduction of zinc (Table 1). Of children with diarrhoea residing in villages with a CHW, use of zinc was much higher at 66% (29/44); however, only nine (5%) of 176 children with diarrhoea in villages without CHWs were reportedly given zinc supplementation in the previous two weeks (p<0.001).

**Table 1:** Reported home management and sources of care visited among young children with diarrhoea in the previous 2 weeks before and 4 months after the introduction of zinc treatment

Reported practices	Baseline: before introduction of zinc (n=228)		Final: 4 months after introduction of zinc (n=220)	
	No.	%	No.	%
Treatments administered*				
Zinc	Not available		38	17.3
Oral rehydration salts	25	11.0	40	18.3
Antibiotics	130	57.0	104	47.3
Metronidizole	17	7.5	8	3.6
Antidiarrhoeal	6	2.6	2	1.0
Home-management practices*				
Sugar salt solution	Question not asked		7	3
Continued feeding or continued breastfeeding	123	54.0	125	56.8
Increased liquids or increased breastfeeding	39	17.1	73	33.2
Source of care visited*				
Community health centre or referral health centre	69	30.3	63	28.6
Community health worker	22	9.7	42	19.1
Unlicensed, informal sector vendor**	100	43.9	78	35.5

*Not mutually exclusive

**Includes market stalls, small shops, and ambulatory vendors

*Data-collection method:* Population-based survey in households with caretakers of sick children

*Population (n):* 228 (baseline survey) and 220 (final survey) children whose caretakers reported having a diarrhoeal illness in the previous 2 weeks

*Timing of data collection:* Before introduction of zinc treatment (April 2004) and 4 months after introduction of zinc treatment (September 2004)

The effect of the introduction of zinc supplementation on care-seeking patterns by families for episodes of childhood diarrhoea was estimated, although definitive conclusions cannot be drawn due to the design of this pilot study. As Table 1 demonstrates, four months after the introduction of zinc, there was little change in levels of care-seeking from the community health centres or from unlicensed, informal sector vendors who commonly sell antibio-tic capsules as a treatment for diarrhoea. However, there was a two-fold increase in reported care-seeking from the CHWs associated with the introduction of zinc supplementation. Even at the conclusion of the study, the CHWs remained a much less common source of care for diarrhoea than the health centres and informal sector providers. This is due partly to the fact that their role in the treatment of malaria has received greater emphasis in recent years than their role in the treatment of diarrhoea, and also because only the larger villages have a CHW.

### Incorporation of zinc into existing diarrhoea-management strategies

The facility-based health workers and CHWs easi-ly incorporated zinc supplementation into their management of childhood diarrhoea. The prescription of the correct dosage of zinc was, overall, satisfactory, with a few difficulties, and is described elsewhere (12). The health centre staff and CHWs were instructed to recommend the purchase of ORS sachets in addition to zinc. In accordance with the Malian Ministry of Health policy of the time, if parents did not or could not purchase the ORS sachets, they were encouraged to prepare sugar salt solution (SSS) in their homes.

The use of ORS and other rehydration therapies was closely monitored in this pilot study. In household follow-up of children treated with zinc at the health centres and by the CHWs (n=123), 64% were prescribed and administered ORS in addition to zinc (Table 2). Of those caretakers who purchased zinc but did not purchase ORS sachets, 62% reported that they were counselled about how to prepare SSS in their homes. Although we do not have comparable data on providers' prescribing practices of ORS before the introduction of zinc, there is little reason to believe that ORS or SSS was recommended at any lesser extent because of zinc. In fact, qualitative data suggest that the providers and CHWs prescribed and emphasized ORS, SSS, and home-fluids more than before because of the training associated with the introduction of zinc and renewed emphasis on management of diarrhoea. In some cases, mothers even reported that the CHWs prepared SSS for them when they indicated that they could not afford to purchase ORS, although the use of SSS reported in the final survey remained low (Table 1). Data from household surveys also suggest that this limited introduction of zinc did not reduce either use of ORS or administration of increased fluids in the home for children with diarrhoea (Table 1). Before the introduction of zinc, only 11.0% of the caretakers reported the use of ORS sachets for the diarrhoea episode of their children in the previous two weeks, an estimate comparable with the most recent DHS survey (18). The proportion of children receiving ORS for diarrhoea four months after the introduction of zinc was 18.3%; a 63% relative increase over the baseline estimates.

**Table 2:** Treatments children received (in addition to zinc) by symptom complex

Reported symptom complex	No.	ORS		Antibiotic		Antimalarial		Paracetamol	
		No.	%	No.	%	No.	%	No.	%
Diarrhoea only	31	15	48	5	16	5	16	3	10
Diarrhoea + fever	36	22	61	1	3	3	8	4	11
Diarrhoea + ARI*	23	18	78	7	30	7	30	3	13
Diarrhoea + ARI* + fever	33	24	73	6	26	6	18	6	18
Total	123	79	64	21	17	21	17	16	13

*Reported symptoms of cough, raised respiratory rate, respiratory difficulties, chest pain, and/or chest in-drawing; ARI=Acute respiratory infection; ORS=Oral rehydration solution

*Data-collection method:* Follow-up survey in household with caretakers of children receiving zinc

*Population (n):* 123 caretakers whose children received zinc from CHWs or health facilities

*Timing of data collection:* 4–12 weeks after the official introduction of zinc treatment

### Non-recommended treatments for diarrhoea and competing sources of modern treatments

The use of modern medications for childhood diarrhoea is extremely high in southern Mali, and many treatments are purchased through the private sector, primarily comprising village markets stalls and ambulatory vendors (19). Almost 44% of children with diarrhoea received treatment from these unauthorized, private providers in the baseline survey (Table 1). Only 3% of children with diarrhoea received an antidiarrhoeal. However, treatment with antibiotics was very common, with 57% of children with diarrhoea reportedly receiving some antibiotics in the baseline survey. While 23% of antibiotics were purchased from the health centre, almost half (49%) was purchased in the market, with another 13% purchased from unauthorized village vendors.

Parents often purchase a few pills at a time at these locations, and during the course of our research, parents frequently expressed their preference of having the flexibility of only purchasing as much or as little medication as they could afford. A CHW, who is recognized by the Ministry of Health, and can provide certain treatments, such as antimalarials, paracetamol, ORS sachets, and also zinc in our study zones, explained the issue as follows:

“The difficulty that we have is the fact that certain people bring only 10 francs or 15 francs to buy medications. If you tell them that you cannot sell them just 10 or 15 francs worth of medication, they get angry and say that, although there is a drug kit, they still cannot get medications … or, as people are used to buying *yalayalafura* [general term for drugs sold in the market or by ambulatory vendors] where there are products for 10–15 francs, they may think that it is the same thing [as medicines from the CHW].”

Although some children likely required antibiotic therapy for co-morbidities or dysentery, evidence showed that parents preferentially sought out antibiotics in cases of uncomplicated diarrhoea, especially from non-authorized providers. Among all sick children (n=352) surveyed at baseline, any reported symptom of diarrhoea was strongly and significantly associated with obtaining antibiotics from an unauthorized source (unadjusted odds ratio=9.4; 95% confidence interval 4.57–19.58) either exclusively or in addition to an antibiotic from a public-health facility. Four months after the introduction of zinc, it was estimated that 47% of children with diarrhoea were treated with antibiotics, an 18% relative decrease from the baseline estimates of antibiotic use.

### Zinc supplementation for diarrhoea and management of children with multiple symptoms

In Mali, children frequently have multiple symptoms concurrently and rarely present with one illness. During the baseline survey, of 352 sick children, approximately 22% reported symptoms of acute respiratory infection (ARI), fever, and diarrhoea, 35% reported fever and diarrhoea, and 17% reported fever and ARI. A similar profile of multiple symptoms was also seen in children who presented for care at community health facilities or CHWs. Table 3 shows that children treated with zinc received from the CHWs were more likely to report diarrhoea as their only symptom than those children treated at the health facilities. However, very few children who were treated with zinc presented with diarrhoea alone in either location, and in both cases, over 70% of children with diarrhoea also had fever, ARI symptoms, or both.

**Table 3:** Symptom patterns of children receiving zinc treatment for diarrhoea at village drug kits or health centres during the zinc pilot study

Reported symptom complex	Total (n=123)		Presenting to village drug kit (n=102)		Presenting to community health centre (n=21)	
	No.	%	No.	%	No.	%
Diarrhoea only	31	25	28	27	3	14
Diarrhoea + fever	36	29	30	29	6	29
Diarrhoea + ARI*	23	19	17	17	6	29
Diarrheoa + fever + ARI*	33	27	27	27	6	29

*Reported symptoms of cough, raised respiratory rate, respiratory difficulties, chest pain, and/or chest in-drawing

Data-collection method: Follow-up survey in household with caretakers of children receiving zinc

Population (n): 123 caretakers whose children received zinc from CHWs or health facilities

Timing of data collection: 4–12 weeks after the official introduction of zinc treatment

The community health centre staff members included nurses who have received training on Integrated Management of Childhood Illness (IMCI), and the CHWs were trained for community-based distribution of medicines for the treatment of malaria. The training modules developed and implemented at the start of the pilot intervention focused almost exclusively on the management of diarrhoea and did not address the management of other childhood illnesses, such as malaria. Despite previous training of health workers on the management of fever, the monitoring of health workers' registers and follow-up of children treated with zinc revealed that many parents of children presenting with both diarrhoea and fever purchased only zinc tablets and ORS sachets, but did not purchase chloroquine (the recommended treatment for all cases of fever at the time of the study). Table 2 presents the treatments given to sick children, in addition to zinc received from the health facilities and CHWs, as reported in household follow-up interviews. These figures also include those treatments obtained from other sources that were given before or after receiving zinc supplementation, as many children receive treatments from various sources of care. For example, in some cases, children with fever and diarrhoea received chloroquine from another source, such as a private vendor, before presenting to a CHW for zinc. Eighteen percent of children with symptoms of diarrhoea, fever, and ARI received an antimalarial; children with symptoms of diarrhoea and fever were even less likely to receive an antimalarial. Treatment of fever with an antipyretic medication (paracetamol) was equally poor.

Due to the difficulties encountered in clinical management of children with fever and diarrhoea, patterns of treatment for children with symptoms of diarrhoea and fever were also further examined through interviews with parents. Although zinc was promoted only for diarrhoea in this study, 20% of 99 caretakers who had heard of zinc interviewed in the final survey stated that zinc can treat malaria, and 9% stated that zinc can treat fever (Fig.). It was also found that community perceptions concerning the effectiveness of modern versus traditional treatments for certain illnesses might play a role in this phenomenon. Previously, children often received only chloroquine for diarrhoea with fever. After the introduction of zinc, when questioned in in-depth interviews, many parents prioritized the purchase of zinc to treat children with diarrhoea and fever. One of the primary reasons was the perception that there are effective traditional treatments for malaria, but not for diarrhoea and that diarrhoea takes away child's strength and, thus, is a more serious illness. As one parent explained:

**Fig.**
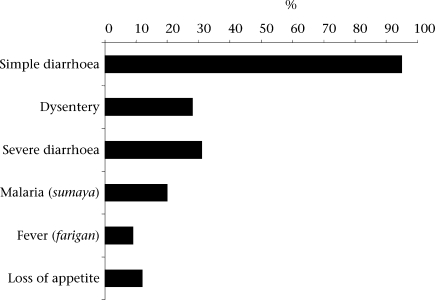
Perceptions of symptoms zinc can treat among 99 caretakers who reported knowledge of zinc

“Malaria (*sumaya*) is easier to treat than diarrhoea. I would begin by treating diarrhoea. There are many treatments for malaria and traditional medicines, which are effective, but for diarrhoea … before, we used *kunbléni* (antibiotic capsules, often tetracycline), but it is not effective, and we mostly use it for severe diarrhoea (*konorojoli*). Therefore, even if I could not buy the malaria medications, I could use the traditional medicines for that.”

### Financial access to zinc and other medications

In qualitative interviews, most caretakers noted that they found zinc affordable, although some caretakers who did not ultimately seek care for their children's diarrhoea stated that the primary reason was due to a lack of funds. In the final household survey, caretakers who reported knowing the price of zinc were asked their opinion concerning its cost. Of 77 caretakers, 72% stated that zinc was inexpensive or affordable, while 6% said that it was expensive or very expensive (22% had no opinion). While the cost of zinc alone may not be prohibitive, when a child presents with multiple illnesses, the total cost of the recommended multiple treatments is not affordable for many families. Before zinc, standard treatment for simple diarrhoea was often traditional remedies or home-fluids supplemented by a few antibiotic pills, frequently tetracycline (commonly called *kunbléni* in Bambara), purchased in the market for a minimal cost (11). Consequently, indivi-dual families spent far more on treating childhood diarrhoea than previously if they administered all the treatments recommended by the project for management of diarrhoea. When diarrhoea was accompanied by fever, the cost of appropriate treatments was even more pronounced.

## DISCUSSION

This study is the first to document the operational issues and examine the trends associated with the introduction of zinc supplementation for diarrhoea in a sub-Saharan setting; previously-published research from Asia concerning community introduction of zinc may be only partially applicable in African settings. Through the evaluation process of the mixed methods employed in this pilot study, we could identify important constraints to the introduction of zinc in this setting, such as the management of concurrent diarrhoea and fever, high use of unauthorized drug vendors, and limited financial access to treatments. We also observed positive trends in key indicators associated with this small-scale introduction of zinc; these include: (a) increased care-seeking to the CHWs for childhood diarrhoea, (b) increased use of ORS, and (c) decreased antibiotic use for childhood diarrhoea. The impact of the larger-scale introduction of zinc on these important indicators are currently being examined in Mali in a community-randomized trial, specifically designed to measure these outcomes.

### Awareness and use of zinc supplementation

The use of zinc supplementation for childhood diarrhoea was much lower than expected four months after the introduction of zinc into the health system. In villages where the CHWs were providing zinc supplementation to young children with diarrhoea, the use of zinc was much higher. Knowledge of zinc supplementation was also significantly better in villages with the CHWs who were engaged in community promotion. We also observed a significant increase in care-seeking to the CHWs for childhood diarrhoea when zinc was introduced. These findings suggest that the CHWs are an effective means for promoting and providing zinc supplementation in communities in this setting, increasing access to and awareness of the new intervention beyond the health centres. However, the relatively low proportion of villages covered by the CHWs in this area (18 villages with Save the Children-supported CHWs among 48 villages and hamlets) limits the potential impact of distribution and promotion of zinc through the CHW system on a population level.

To improve the awareness and use of zinc supplementation at a population level, village drug kits managed by trained CHWs could be established in all villages further than five km from a community health centre. This would require significantly more funding and programmatic effort, as villages tend to be dispersed, so supervision and support of CHWs would be more expensive. Distributing zinc through private vendors of modern medicines is another alternative, especially in this setting where vendors are a common source of treatment for childhood diarrhoea. Despite the elevated use of these providers, zinc was not provided via this system in this pilot for the following reasons: (a) very few private outlets or vendors are sanctioned by the Government; (b) many vendors are without a fixed place of work; (c) some vendors are children as young as 8 or 9 years of age; (d) it is difficult to monitor side-effects and outcomes of zinc treatment obtained from these sources of care; and (e) it is difficult to assure the quality of the product, as the sources of medications in the informal sector have not yet been determined. However, in the future, private providers and/or vendors might be involved in the distribution of zinc. Because zinc is a micronutrient with no chance of evolving resistance and has very few documented side-effects, obtaining government approval for such an approach may be less complicated than efforts to gain permission for distributing antimicrobials, such as antimalarials, in the private sector.

Additional channels of communication should also be used for reaching desired levels of coverage. Other evaluations in Africa have found social marketing using mass media to be an effective means of health promotion (20), although where communities are dispersed and have limited access to modern media, coverage achieved through national media alone will be insufficient (20-22). In this area, community-based promotion of zinc as a new treatment could include broadcasts on local radio stations and promotion by health centre staff at vaccination days at the health centre, during antenatal visits, or when facility-based nurses make monthly visits to villages for vaccination, distribution of vitamin A, and other public-health activities. Suggestions given by the CHWs and project staff for other strategies appropriate to the Malian context include: (a) plays created by theatre groups, (b) drumming and/or dancing with the zinc messages, (c) messages about zinc broadcast during village special events, (d) information taught in schools so students will pass along information to their parents, and (e) skits developed and performed by school students in the villages.

### Incorporation of zinc into current diarrhoea-management practices

Although this pilot phase of the study was not designed to measure changes in the use of ORS, the results suggest that introducing zinc into the management of diarrhoea may improve the use of ORS, a result similar to that found in Bangladesh and India (8,10). We also observed that the introduction of zinc supplementation did not reduce administration of increased fluids in the home; in fact, there was an increase in recommended home care associated with the introduction of zinc supplementation.

The Ministry of Health, Mali, now no longer re-commends the use of SSS. In accordance with the recommendations of WHO and UNICEF (5), the Ministry of Health has revised their policy to promote increased appropriate home-fluids (and continued feeding) for children with diarrhoea. Because the administration of appropriate home-fluids is simpler to explain and carry out than the preparation of SSS, it is hoped that this new approach will improve both promotion of ORT by health workers during consultations and subsequent administration of ORT by parents in the home. The new low-osmolarity ORS formulation, which has been shown to reduce stool output, vomiting, and unscheduled intravenous infusions (23-25), is not yet available in Mali. When this new ORS formulation becomes available, health workers should promote this product along with zinc to families as an improved, more efficacious diarrhoea-management package.

### Non-recommended treatments for diarrhoea and competing sources of modern treatments

In Mali, many parents perceive that childhood diarrhoea is better treated with antibiotic capsules, and purchase of antibiotics from the informal sector for childhood diarrhoea is extremely common. In this setting, zinc has a great potential to replace antibiotics as a ‘medicine' for diarrhoea, thereby decreasing overall antibiotic use among children. Our evidence points to a slight decrease in antibiotic use and a small decrease in care-seeking to unauthorized medicine vendors associated with the introduction of zinc supplementation. These results are comparable with a recent study in Bangladesh which found that 13% of children with diarrhoea living in zinc clusters were being treated with antibiotics for their diarrhoea episodes compared to 34% of children with diarrhoea living in control clusters (p<0.01) (8). To reinforce these trends, promotional messages accompanying introduction of zinc should discourage antibiotic use for non-dysenteric cases of diarrhoea. Because parents are accustomed to purchasing and administering a few pills or capsules from market vendors for an episode of diarrhoea, the longer-term preventative aspects of zinc supplementation (4,9), and, thus, the potential reduction in future medicine purchases, should also be emphasized in these promotional activities.

### Zinc supplementation for diarrhoea and management of children with multiple symptoms

Insufficient attention devoted to the management of multiple symptoms during the training of CHWs and health centre staff was the largest operational issue encountered by our study team. Within two weeks of noting the deficiency in management of children who present with fever and diarrhoea, day-long meetings were held, in cooperation with the local health authorities, to address this shortcoming of the intervention. It was decided to provide follow-up ‘refresher' training to the CHWs. Discussions during this training and subsequent interviews with the CHWs revealed that many of them had been working under the misconception that the combination of treatments was not nece-ssary. During the refresher training, the CHWs stated, “we did not know that it is necessary to also treat malaria; we believed that treatment of diarrhoea was sufficient,” and asked whether, “the child could handle two treatments at once,” reflecting local perceptions that some medicines and medicine combinations are too powerful for children. A review of drug kit registers (notebooks) and a second series of interviews after the refresher trainings confirmed that the CHWs were appropriately managing children presenting with diarrhoea and fever.

Health programmes must take two basic steps to ensure adequate management of children with multiple conditions. First, programmes must ensure that health workers treat all the children's conditions adequately. Zinc must be incorporated into an integrated package of disease management and cannot be introduced in isolation. Training must also cover management of common combinations of presenting symptoms, such as diarrhoea and fever, or diarrhoea and ARI. Job aids and registers/notebooks for recording symptoms and treatments should be designed with the presentation of multiple symptoms taken into consideration. Ade-quate management of all symptoms will likely be facilitated in areas implementing Integrated Management of Childhood Illness (IMCI), which has been shown to improve the quality of care for sick children in facilities (26).

Second, programmes must ensure that parents purchase and administer all the required medications for children presenting with multiple symptoms. This is a particular concern as Mali makes the transition to artemisinin combination therapy (ACT) as the first-line treatment for malaria. If zinc and ACTs are introduced concurrently, this may compound the challenge of adequately explaining how to manage sick children to parents and first-level and voluntary health workers, as both are new treatments for common childhood illnesses. A child presenting with diarrhoea and fever may be pres-cribed an antimalarial, an antipyretic, zinc, and ORS. Health workers will require more detailed counselling guidelines for explaining the importance of purchasing multiple treatments and how to administer various medications.

### Financial access to zinc and other treatments

The introduction and promotion of zinc supplementation, along with improved management of diarrhoea, results in families spending more on treatment of diarrhoea than they are accustomed. This will force poor families to make difficult choices—which one of the life-saving treatments recommended for their sick child will they purchase? This study has demonstrated that when a new treatment, such as zinc supplementation for diarrhoea, is introduced, although its cost is minimal, parents may try to judge which treatment is the most important and purchase only that one treatment. When multiple treatments, such as antimalarials, zinc, and ORS, are prescribed, the poorest families may gravitate to the lowest treatment cost. Given the high cost of new combination treatments for malaria, there is a real possibility that many families will purchase zinc instead of antimalarials when their children present with fever and malaria. If new combination treatments for malaria are highly subsidized or free to parents, there is the possibility that children presenting with fever and diarrhoea may only receive an antimalarial.

It is increasingly recognized that there is a moral imperative for governments and donor organizations to make more expensive life-saving treatments, such as antiretroviral therapy for AIDS and artemisinin combination therapy for malaria, available free of charge or at a minimal price to those who are in desperate need, but could never afford to pay for them. A similar consensus in support of subsidizing treatments for diarrhoea has yet to be materialized. One reason is that low-osmolarity ORS and zinc in a sense are affordable. Families could borrow from a neighbour or sell some of their belongings to purchase ORS and zinc recommended for their sick children, and intensive marketing of zinc might convince them to do this on a regular basis. But this is a dangerous zero-sum game. Improved management of diarrhoea may mean inadequate management of other illnesses or decreased spending on food. The experience from this study in Mali suggests that families who are carefully husbanding their scarce resources often will fail to purchase and administer all the recommended treatments for their sick children, when multiple treatments are recommended. We believe that there is a compelling case for governments and donor organizations making the commitment to ensure that zinc is available free of charge or at a nominal cost, if we hope to see its life-saving potential realized.

For children with multiple symptoms, policy-makers need to ensure that there is no financial penalty for purchasing multiple essential treatments for sick children. As new drugs are introduced for diarrhoea, malaria, and other illnesses, policy-makers need to examine the costs that will be incurred when different combinations of symptoms are treated and develop policies for subsidization that will not only make expensive medications, such as artemisinin combination therapy affordable, but also promote rational treatment behaviour for children with multiple symptoms.

In Mali and in similar health systems based on cost-recovery, credit is often extended to families in the community who cannot afford to pay for medications recommended by the CHWs, especially during the rainy season. When credit is extended but never repaid, the CHWs are still responsible for replenishing their supplies and at times have to use personal funds to purchase new stocks and/or to obtain transportation to the health centre to purchase these stocks. In a region where personal funds are often extremely limited, this can lead to stock outs. Problems relating to financial access not only impact individual families, but can also negatively impact the distribution systems. Therefore, decisions about total or partial subsidization of zinc and ORS need to consider not only how to ensure financial access to these treatments for the poorest families, but also how to ensure the sustainability of community-based distribution systems that rely on voluntary or semi-voluntary workers, such as CHWs.

Our findings demonstrate that factors, such as management of concurrent diarrhoea and fever, high use of unauthorized drug vendors, and financial access to treatments are crucial factors affecting the effectiveness of zinc treatment when introduced into routine programmes. Preliminary evidence from this pilot study suggests that the introduction of zinc does not reduce the use of ORS; in fact, it has the potential to reinvigorate existing diarrhoea-management strategies and to reduce inappropriate antibiotic use for simple diarrhoea. A community-based cluster randomized trial is currently underway to examine the impact of the introduction of zinc on diarrhoea-management practices, such as use of ORS, seeking care at the community health centres and CHWs for childhood diarrhoea and antibiotic use for simple diarrhoea.
